# Youth Football Tournaments: Are We Developing Players or Harming Their Growth?

**DOI:** 10.7759/cureus.83146

**Published:** 2025-04-28

**Authors:** Diogo Roxo, Francisco Tavares, Nuno Loureiro, Jorge Fortunato, Ruben Ferreira, João Pedro Araújo

**Affiliations:** 1 Physical Medicine and Rehabilitation Department, Hospital de Cascais, Lisbon, PRT; 2 Medical and Performance Department, Sporting Clube de Portugal, Lisbon, PRT

**Keywords:** external load, footballers, growth, injury, maturation

## Abstract

Currently, many children and teenagers dream of becoming football players and dedicate a large part of their time to achieving this goal. The clubs’ medical and performance departments' primary mission is to reduce the risk of injury to these athletes, allowing them to have more playing and training time to develop their full capabilities. This study aimed to investigate whether participation in youth football tournaments with congested schedules increases the risk of injury in U15 footballers, particularly during critical stages of maturation. In this way, in a retrospective manner, an analysis was conducted of the injuries recorded after a period marked by the participation of a U15 elite team in a preseason youth football tournament. Additionally, the maturational stage of each athlete on this team was recorded and the external workload data through the Global Positioning System (GPS), which included total distance (TD), high-speed running (HSR), accelerations and decelerations greater than or less than 2 m·s^−2^ (ACC-DEC2), and accelerations and decelerations greater than or less than 3 m·s^−2^ (ACC-DEC3), from the weeks before the tournament were used, aiming to understand whether, in the injured athletes, the accumulation of these metrics during the tournament exceeded the accumulation of the same metrics before the tournament. According to the results obtained, eight athletes sustained injuries during the tournament and the two weeks following it. All injured athletes were in the circa or post-peak height velocity (PHV) maturation stage. In the analysis of the GPS data, it was found that 87.5%, 75%, and 50% of the athletes who suffered an injury exhibited higher rolling sum (RS) values during the tournament compared to their previously recorded maximum values regarding TD, HSR, and ACC-DEC2, respectively. In contrast, 75% of the athletes who did not suffer an injury recorded higher maximum RS values before the tournament compared to during the tournament regarding the parameter of ACC-DEC3. The participation of young footballers in the circa-PHV and post-PHV stages of maturation in several games on consecutive days seems to induce negative consequences for the athletes, namely a greater risk of overuse and acute injuries. The external load metrics presented appear to correlate with the risk of injury, specifically a higher risk of injury in athletes whose accumulated values of TD, HSR, and ACC-DEC2 during competition participation are higher than those previously recorded. Conversely, the presence of higher accumulated values of ACC-DEC3 prior to competition participation compared to those recorded during the competition may be a protective factor regarding injury risk. Careful management of microcycles and applied workloads is a key task to control or prevent injuries and, in this way, increase the young footballers’ participation in games and training.

## Introduction

Football’s global popularity and extensive media coverage inspire most children to dream of becoming football players. Moreover, the high process of player transfers, constant media coverage, and the frequent appearance of football players in advertisements heighten expectations not only among children and teenagers but also among their parents, and later, among football agents and clubs seeking to maximize the value of their assets [1].

The integration of athletes into training academies aims to accelerate the training process and enhance their capabilities. Athletes who dedicate a significant portion of their time to practicing football rely on the invaluable support of clubs' medical and performance teams to minimize the risk of injury [1,2].

Raysmith and Drew found that young athletes who complete more than 80% of their training weeks are seven times more likely to achieve their goals [3]. For this to happen, athletes must maintain a balance between training load and recovery [2], as an imbalance favoring either can lead to illness or injury [4]. Both monitoring loads, through internal or external measures, and monitoring athletes' recovery are part of the daily routine of football clubs [5], and this work is essential to maximize performance in the next game [6].

Nowadays, there are several obstacles regarding the recovery of athletes, such as the high number of games or tournaments, as well as an overloaded calendar. In adult elite football players, Dupont et al. and Lago-Peñas et al. suggest a period of 72-96 hours after the game to once again achieve a stress-recovery balance similar to the pregame [7,8]. For his part, regarding college-level American footballers with an average age of 20.6 years, Fullagar et al. suggest a period of 96 hours [9].

Youth football teams frequently participate in tournaments, particularly during periods of competitive break. These tournaments are characterized by their congested calendars as they require games on consecutive days and sometimes more than one game on the same day. Mandorino et al. [4], Brink et al. [10], Bacon and Mauger [11], and Bowen et al. [12] demonstrated in previous studies that exposure to high acute loads in athletes subjected to low chronic loads was related to an increased incidence of injuries.

The present study focuses on comparing the accumulated values ​​of external load among players of an elite U15 team during an intense competitive period (a three-day preseason youth football tournament with games on consecutive days) and during the preparation period for it (three weeks before participation in the aforementioned tournament). The primary objective is to establish a correlation between the external loads to which athletes are exposed and an increased incidence of injury. Other objectives include trying to determine the existence of some external load parameters that can be considered a protective factor against injury and the description of the maturity stage of the athletes included in the study. Considering previous literature, an increased risk of injury is attributed to young athletes, namely in the circa-peak height velocity (PHV) and post-PHV stages of maturation, who are subjected to high loads [4,6].

## Materials and methods

Study design and participants

This article consists of a descriptive and observational study in which a retrospective analysis was carried out using the Global Positioning System (GPS) data of an elite U15 team at Sporting Clube de Portugal’s training academy. Data were obtained during the first three weeks of preseason, between July 22, 2024, and August 11, 2024, which culminated with the participation of the team in a youth football three-day tournament. Additionally, a retrospective analysis was also carried out on the number and type of injuries and illnesses recorded during the tournament and in the following two weeks, between August 9, 2024, and August 25, 2024. The inclusion criteria were players from Sporting Clube de Portugal’s U15 team and players with GPS data available. The exclusion criteria were goalkeepers, players who were not called up for the tournament, players with previous injuries during the study period, and incomplete data.

A total of 29 elite football players from the U15 team were recruited to participate in this study. According to the exclusion criteria, the final sample was 16 players. Given the impositions of the tournament organization about the limit of players participating per team (i.e., 18 players), 11 athletes were immediately excluded. The two goalkeepers called up for the tournament were also excluded, as there are no available data on external load for athletes in this position. The absence of GPS data in these athletes is due to its limited relevance, as activities involving speed, distance coverage, or acceleration are minimal in this position during sports practice.

Each player was randomly assigned a number from 1 to 16 to protect their personal information when presenting the results, particularly regarding the details of injuries sustained during the study period and the data collected via the GPS device.

Injuries

During the tournament and in the two weeks following it, all injury and illness situations that justified the athlete's absence from any training or game period were recorded. The period, out of competition, that resulted from each injury and illness was also recorded. Injuries were classified according to Brukner and Khan’s Clinical Sports Medicine classification of sporting injuries: overuse injuries and acute injuries that include direct/contact mechanism and indirect/noncontact mechanism injuries [13]. The diagnosis of injuries was consistently performed by the same team of medical professionals, thereby eliminating potential interpersonal confounding factors. The assessment followed a specific protocol, which included visualizing the injury mechanism either at the time of occurrence or via video, conducting an anamnesis with the athlete, performing an objective examination focused on the complaints, and carrying out additional diagnostic tests, such as ultrasound or radiography. After diagnosing the injury, the medical team defined an individualized rehabilitation program that could involve physiotherapy and progressive adjustments to workloads. Overuse injuries include apophysitis, tendinopathies, chondropathies, bone stress fractures, or delayed onset muscle soreness. Direct mechanism injuries include bone fractures, periosteal contusions, and muscle contusions, while indirect mechanism injuries include muscle injuries such as strains or tears, tendon tears, or joint sprains. Finally, illnesses like the flu or acute gastroenteritis were also recorded. Injuries and illness were also recorded during the two weeks after the tournament, given the delayed effect that training load has on injury occurrence [14].

Anthropometry and body composition

The anthropometry assessment was carried out by an experienced technician, certified by the International Standards for Anthropometric Assessment (ISAK) Level I, who performed anthropometric measurements (body mass, standing height, and sitting height) following the guidelines of the International Society for the Advancement of Kinanthropometry [15]. Standing height and sitting height were measured with an anthropometer (SECA 206) with an accuracy of 0.1 cm, and weight was measured with an electronic scale (TANITA BC-601) with an accuracy of 0.1 kg. Eight skinfolds (8SKF), such as biceps, triceps, subscapular, suprailiac, supraspinal, abdominal, front thigh, and medial calf, were collected using a Harpenden adipometer (British Indicators, Ltd., London, United Kingdom). Each location was measured twice, with an accuracy of 0.1 mm. Dual anthropometric measurements were obtained for each site, and the average of the two measurements was used to calculate the 8SKF. An intraobserver measurement error of 5% was considered acceptable for the skinfolds. Otherwise, a third measurement was performed.

Height, body mass, chronological age, and average height of the parents were used for the predicted adult height (PAH) of each player and to verify their biological maturity [16]. The heights of each player's biological parents were self-reported and adjusted for overestimation using the equations by Epstein et al. [17]. Therefore, athletes were classified according to maturity status using percentage of PAH: circa-PHV represents 88%-96% of PAH, pre-PHV represents <88% of PAH, and post-PHV represents >96% of PAH [18-20]. The use of the Khamis-Roche method [16], instead of other methods such as the Tanner-Whitehouse method, is justified by the fact that it does not require skeletal X-rays, making it safer, quicker, and easier to apply in field settings such as youth football academies. Moreover, it is more suitable for use during mass screenings or routine assessments of youth athletes. While other methods may be accurate, they are often time-consuming, expensive, and invasive.

GPS data collection/external payload

During each training session, players were continuously monitored with the same GPS unit (APEX, StatSports, United Kingdom), with the device placed on each player's upper back using an adjustable neoprene harness to do so. The GPS units were turned on before warm-up and turned off after the training and game sessions were completed. After each session, the raw data files were analyzed. Their corresponding metrics, total distance (TD), high-speed running (HSR), accelerations and decelerations greater than or less than 2 m·s^-2^​​​ (ACC-DEC2), and accelerations and decelerations greater than or less than 3 m·s^-2^ (ACC-DEC3), were obtained from STATSports Sonra Analysis Software (STATSports, Newry, Ireland). Speed data were collected at 10 Hz, with the signal of 16 ± 2 satellites.

Data collection for this retrospective analysis was carried out within the typical training and game routines of the level, under the supervision of the usual technical and medical committee. All procedures were conducted in accordance with the Declaration of Helsinki.

GPS data analysis/external load

From the range of GPS data collected for each athlete, and in line with previous studies [11], four parameters were selected for analysis: TD in meters (m), HSR in meters, ACC-DEC2 in meters, and ACC-DEC3 in absolute frequency (n). For speed parameters, HSR was included, representing the sum of distances covered by an athlete at speeds exceeding 5.5 m·s (19.8 km·h). Accelerations were recorded when an athlete’s speed increased by more than 2 or 3 m·s^-2^ for a duration exceeding 0.5 seconds, while decelerations were recorded in a similar manner. The composite parameters ACC-DEC2 and ACC-DEC3 were calculated by summing the respective accelerations and decelerations.

To further explore the data and investigate whether the load accumulated by athletes over consecutive days was related to injury incidence, statistical analysis was performed using the rolling sum (RS) of these metrics over three-day, five-day, and seven-day periods. The RS values were calculated by summing the daily GPS data for each metric, enabling the determination of accumulated external load. As an example, to obtain the RS over a three-day period for the TD parameter, the sum of the TD values for each day and the two preceding days was calculated for each athlete during the three weeks of preseason in which GPS data were collected, culminating with the participation in a youth football three-day tournament. The same procedure was applied to the remaining parameters. Since the tournament lasted three days, this period was used to calculate the RS. RS values for five- and seven-day periods were also analyzed to assess the accumulation of external loads over a typical workweek. This approach facilitated a comparison between the maximum RS recorded during the tournament and the highest RS observed prior to the tournament.

## Results

During the tournament and the two weeks following it, eight athletes sustained injuries. These included one direct mechanism injury, four indirect mechanism injuries, and three overuse injuries. No illnesses were reported during this period. Additional details, such as the type of injury, the date of occurrence, the clinical discharge date, the date of full reintegration with the team (return to play without limitations), and the total time of incapacity (absence days), are presented in Table 1. From the data in this table, it can be concluded that 50% of the injured athletes (four athletes) were in the circa-PHV maturation stage, while the remaining 50% (four athletes) were in the post-PHV maturation stage.

**Table 1:** Characterization of the type of injury, absence days, and maturation stages of athletes who suffered injuries since the tournament until the two weeks following it

Player	Date of injury	Type of injury	Clinical discharge	Return to play without limitations	Absence days	Maturation stage (Khamis-Roche method)
9	August 9, 2024	Indirect mechanism of injury	August 16, 2024	September 4, 2024	26 days	95.4%
8	August 9, 2024	Direct mechanism of injury	August 20, 2024	August 24, 2024	15 days	94.6%
11	August 10, 2024	Indirect mechanism of injury	August 22, 2024	September 4, 2024	25 days	96.5%
16	August 10, 2024	Indirect mechanism of injury	August 14, 2024	August 20, 2024	10 days	95.8%
12	August 17, 2024	Overuse injury	August 28, 2024	August 31, 2024	14 days	96.6%
6	August 20, 2024	Overuse injury	August 22, 2024	August 31, 2024	11 days	98.4%
5	August 20, 2024	Overuse injury	August 27, 2024	August 31, 2024	11 days	95.6%
13	August 24, 2024	Indirect mechanism of injury	September 17, 2024	September 24, 2024	31 days	96.5%

In Tables 2-5, we can find the RS of three, five, and seven days of several parameters, namely TD, HSR, ACC-DEC2, and ACC-DEC3, respectively. RS results present the sum of the accumulated values for each metric over a period of three, five, or seven days.

**Table 2:** Characterization of RS values of three (RS3), five (RS5), and seven (RS7) days of TD by athletes ^*^Players who were injured ^**^Players who suffered injury and whose maximum RS during the tournament was greater than their maximum RS leading up to the tournament TD: total distance; RS: rolling sum

Player	TD (m)					
	RS3		RS5		RS7	
	Maximum during the tournament	Maximum until the tournament	Maximum during the tournament	Maximum until the tournament	Maximum during the tournament	Maximum until the tournament
1	27,197	19,433	38,419	28,922	43,534	32,692
2	20,951	20,359	33,605	28,821	37,894	33,866
3	22,685	14,310	33,007	20,545	36,995	25,335
4	18,071	18,027	27,918	22,164	32,365	30,386
5^*^	14,435	17,234	25,364	26,653	30,102	30,229
6^*^	20,475^**^	19,150^**^	31,190^**^	30,169^**^	35,887^**^	31,659^**^
7	10,752	16,477	20,823	23,183	22,227	26,897
8^*^	22,566^**^	19,474^**^	32,011^**^	26,598^**^	36,408^**^	29,931^**^
9^*^	19,906^**^	17,691^**^	27,309^**^	24,993^**^	31,382^**^	29,209^**^
10	22,270	18,785	33,758	27,236	38,655	31,875
11^*^	23,369^**^	19,129^**^	32,122^**^	27,943^**^	36,899^**^	31,590^**^
12^*^	27,678^**^	21,220^**^	38,805^**^	29,563^**^	43,958^**^	33,936^**^
13^*^	24,593^**^	20,406^**^	35,638^**^	28,909^**^	40,508^**^	33,115^**^
14	19,255	19,251	31,463	23,991	33,997	31,459
15	18,345	18,280	27,390	26,098	31,487	31,585
16^*^	17,728	17,767	28,865^**^	28,237^**^	32,354^**^	29,401^**^

**Table 3:** Characterization of RS values of three (RS3), five (RS5), and seven (RS7) days of HSR by athletes ^*^Players who were injured **Players who suffered injury and whose maximum RS during the tournament was greater than their maximum RS leading up to the tournament HSR: high-speed running; RS: rolling sum

Player	HSR					
	RS3		RS5		RS7	
	Maximum during the tournament	Maximum until the tournament	Maximum during the tournament	Maximum until the tournament	Maximum during the tournament	Maximum until the tournament
1	1,826	1,166	2,316	1,831	2,543	1,831
2	1,397	777	1,708	1,189	1,810	1,189
3	1,189	414	1,475	588	1,542	759
4	767	1,027	1,168	1,391	1,639	1,609
5^*^	573	862	802	1,238	1,022	1,238
6^*^	789	862	1,107	1,337	1,232	1,363
7	359	603	513	893	621	893
8^*^	459	469	657^**^	646^**^	677	687
9^*^	381	885	502	1,283	935	1,283
10	946	733	1,147	1,089	1,175	1,089
11^*^	1,137^**^	830^**^	1,531^**^	1,233^**^	1,630^**^	1,385^**^
12^*^	1,264^**^	872^**^	1,534^**^	1,233^**^	1,671^**^	1,294^**^
13^*^	1,905^**^	1,300^**^	2,379^**^	1,919^**^	2,581^**^	1,919^**^
14	1,076	1,144	1,384	1,396	1,404	1,446
15	1,046	966	1,588	1,488	1,645	1,489
16^*^	805	893	1,170	1,350	1,274	1,350

**Table 4:** Characterization of RS values of three (RS3), five (RS5), and seven (RS7) days of ACC-DEC2 by athletes *Players who were injured ^**^Players who suffered injury and whose maximum RS during the tournament was greater than their maximum RS leading up to the tournament ACC-DEC2: accelerations and decelerations greater than or less than 2 m·s^-2^; RS: rolling sum

Player	ACC-DEC2 (m)					
	RS3		RS5		RS7	
	Maximum during the tournament	Maximum until the tournament	Maximum during the tournament	Maximum until the tournament	Maximum during the tournament	Maximum until the tournament
1	5,058	4,521	7,036	6,797	8,144	6,968
2	3,721	3,652	5,628	5,266	6,261	5,722
3	4,199	2,958	5,870	3,534	6,641	4,399
4	2,890	2,948	4,661	3,679	5,481	5,096
5^*^	2,079	2,980	3,628	4,726	4,374	4,726
6^*^	3,003	3,196	4,501	4,872	5,266^**^	5,003^**^
7	1,863	2,879	3,344	3,972	3,643	4,437
8^*^	2,725^**^	2,589^**^	4,018^**^	3,541^**^	4,567^**^	3,997^**^
9^*^	2,539	2,904	3,674	4,257	4,470^**^	4,345^**^
10	3,192	3,078	4,564	4,320	5,329	4,752
11^*^	3,624^**^	3,249^**^	4,903^**^	4,595^**^	5,767^**^	5,238^**^
12^*^	4,663^**^	3,412^**^	6,380^**^	4,663^**^	7,403^**^	5,433^**^
13^*^	4,818^**^	4,193^**^	6,873^**^	6,049^**^	7,989^**^	6,797^**^
14	3,577	3,786	5,274	4,703	5,845	5,660
15	2,923	3,449	4,568	4,861	5,394	5,752
16^*^	3,067	3,832	4,785	5,938	5,417	5,938

**Table 5:** Characterization of RS values of three (RS3), five (RS5), and seven (RS7) days of ACC-DEC3 by athletes ^*^Players who were injured ^**^Players who suffered injury and whose maximum RS during the tournament was greater than their maximum RS leading up to the tournament ACC-DEC3: accelerations and decelerations greater than or less than 3 m·s^-2^; RS: rolling sum

Player	ACC-DEC3 (n)					
	RS3		RS5		RS7	
	Maximum during the tournament	Maximum until the tournament	Maximum during the tournament	Maximum until the tournament	Maximum during the tournament	Maximum until the tournament
1	518	584	718	795	859	865
2	325	412	526	548	576	573
3	370	321	528	358	625	420
4	259	256	438	343	500	458
5^*^	196	336	357	465	432	477
6^*^	237	320	380	448	453	470
7	182	323	328	400	360	461
8^*^	223	291	353	378	404	423
9^*^	197	248	305	382	353	401
10	218	275	327	387	371	399
11^*^	330^**^	321^**^	456^**^	452^**^	533^**^	489^**^
12^*^	376^**^	356^**^	602^**^	492^**^	708^**^	599^**^
13^*^	477^**^	439^**^	700^**^	620^**^	849^**^	729^**^
14	315	371	496	474	535	580
15	261	329	427	482	497	560
16^*^	265	444	477	592	530	642

In the analysis of TD data, 87.5% of injured athletes (seven out of eight) exhibited higher RS values during the tournament compared to their previously recorded maximum values. Player 16, who displayed this trend only in RS5 and RS7, was included in this result.

For HSR values, it was observed that 50% of the injured athletes (four out of eight) recorded higher maximum RS values during the tournament than before it. Player 8, whose results showed this trend only in RS5, was also included in this category.

Regarding ACC-DEC2 values, 75% of the injured athletes (six out of eight) exhibited higher maximum RS values during the tournament compared to their prior maximums. Players 6 and 9 were considered part of this group, although their higher values during the tournament were only observed in RS7.

In terms of ACC-DEC3 values, only 37.5% of injured athletes (three out of eight) achieved higher maximum RS values during the tournament than before it. The remaining athletes had already recorded higher RS values in earlier weeks compared to those during the tournament.

Among the eight athletes who were not injured during the study period, an analysis of the ACC-DEC3 parameter revealed that 75% (six out of eight) recorded higher maximum RS values before the tournament compared to during the tournament. Players 2 and 14 were included in this group, although their higher pretournament values were observed in only two of the three RS metrics.

For the other parameters, TD, HSR, and ACC-DEC2, this pretournament superiority in RS values was observed in 12.5%, 37.5%, and 25% of the athletes, respectively.

## Discussion

This study investigated the external loads that U15 athletes were subjected to during a three-week period that culminated with participation in a three-day tournament. In this way, an attempt was made to establish a correlation with the occurrence of injury or illness.

The tournament in question occurred during the preseason of the studied team and brought together some of the best teams on the Iberian Peninsula at the U15 level. In addition to only 16 field players being able to be called up for the three days of the tournament, each team played a total of three games (one game per day on consecutive days), with each game lasting 70 minutes, which in of itself constitutes a period of a high competitive overload, particularly in a preparatory stage where resilience to loads is lower. This was concluded by Brito et al. at the U15 level in Portugal, who described a total incidence of 2.7 injuries per 1,000 hours of exposure during the preseason, instead of 1.1 injuries per 1,000 hours of exposure during the season [21,22]. The preseason period should be characterized by a progressive increase in training loads to increase tolerance and resilience, controlling the risk of injuries.

In young football players, the risk of injury is high as they are exposed to elevated external loads and high levels of psychosocial stress, which impact recovery [2,10], as well as the maturation stage they are in [4,6,23]. In particular, the spontaneous increase in growth rate, described as PHV, appears to be associated with the most critical period. PHV is commonly used to define the maturation stage of athletes, and although many equations can be used, the Khamis-Roche method is the most frequently utilized [16]. Maturation stages refer to the physical, hormonal, and biological development processes during puberty, occurring at different times in athletes, regardless of their chronological age. Most studies classify athletes as pre-PHV (up to 88% of estimated height), circa-PHV (between 88% and 96%), and post-PHV (above 96%) [1,18,24]. Mandorino et al. concluded that athletes in the circa-PHV and post-PHV stages experience higher injury rates and impacts compared to pre-PHV athletes, while also demonstrating a lower recovery capacity [4,6]. Pre-PHV athletes have an injury risk approximately three times lower than that of a circa-PHV athlete [23]. According to the obtained results, out of the eight athletes who suffered injuries, four were in the circa-PHV period and another four were in the post-PHV period. Overall, of the 29 athletes that make up the U15 team's squad, 65.5% were in the circa-PHV stage, 31% in the post-PHV stage, and 3.5% on the pre-PHV stage. Regarding the athletes' maturation stages, the risk of injury in this team was increased ad initium given that most of the athletes were in the circa-PHV and post-PHV maturation stages. This risk increases with the possible excessive load imposed on players by overloaded competitive periods, as happened with participation in this tournament.

Regarding the training loads the players were exposed to, the study benefited from the fact that all athletes on this team had their data collected since the beginning of the season using a GPS system, an uncommon practice for athletes under the age of 16 [1].

During the study period, three overuse injuries were recorded: players 5, 6, and 12. In the case of player 5, no RS values were recorded during the tournament that were higher than the RS values before the tournament. Regarding player 6, this significant difference was just obtained in relation to TD. In the case of player 12, supramaximum RS values were obtained during the tournament in all analyzed parameters: TD, HSR, ACC-DEC2, and ACC-DEC3. Regarding the incidence of overuse injuries, Mandorino et al. had already concluded that an exponential rise in acute loads poses a greater risk of overuse injuries, particularly in athletes with lower previous loads over preceding days and weeks [4]. Bacon and Mauger concluded that the values of TD constitute a good predictor of the risk of overuse injury [11], which is in line with the results we obtained regarding injuries in players 6 and 12.

About acute injuries, there was only one direct mechanism of injury: player 8. This injury can be associated with exposure to higher acute loads, namely TD and ACC-DEC2. This result is in line with the results obtained by Brink et al., who established that athletes exposed to higher loads face an increased risk of direct mechanical injuries [10]. Also, Bowen et al. had already settled that the high acute/chronic ratios of TD and ACC were associated with a higher risk of direct mechanism injury [12]. On the other hand, there were a total of four indirect mechanism injuries, players 9, 11, 13, and 16, which resulted in longer total times of incapacity (absence days). Two of these four athletes obtained maximum RS values during the tournament that were higher than the RS values recorded up to the tournament in the four external load parameters included in this study: TD, HSR, ACC-DEC2, and ACC-DEC3. With regard exclusively to the recorded TD values, all four athletes who suffered indirect mechanism injuries presented supramaximum RS values during the tournament compared to the values recorded until then. Bacon and Mauger had already associated a large accumulation of TD, HSR, and ACC with a greater probability of injury [11]. On the other hand, Bowen et al. concluded that a high acute HSR distance, combined with a low chronic HSR distance, substantially increases the risk of indirect mechanism injury [12].

Regarding HSR values, Carmona et al. investigated and established a direct relationship between high values of this metric and the presence of postgame changes in muscle distress markers such as creatine kinase levels or in performance tests such as the counter movement jump [25]. Considering hamstring muscle injuries, this muscle group ends up being subjected to a large number of eccentric contractions in the sprint during the game, resulting in muscle damage. Carmona et al. determined that, when compared with the pregame samples, 72 hours after the effort of the game, there was still a statistically significant: increase in disruptions of muscle fibers in the long head of the biceps femoris; a decrease in joint range of motion of this muscle group, due to an increase in passive muscle tension induced by muscle damage; and a decrease in hamstring strength when performing isometric knee flexion [25].

According to the results, there is a high preponderance of the fact that maximum RS values were reached during the tournament regarding the TD, HSR, and ACC-DEC2. This shows that the high loads to which athletes were subjected during these three days may have been decisive for the occurrence of injury. The existence of an injury results in less use and participation in games and training, which, consequently, has a disastrous effect on the development of technical, tactical, physical, and psychological skills [1]. Ultimately, it can lead to stagnation in the athlete's football development and dismissal from the club [4].

According to the results presented, it was found that 87.5%, 75%, and 50% of the athletes who sustained an injury exhibited higher RS values during the tournament compared to their previously recorded maximum values regarding TD, HSR, and ACC-DEC2, respectively. Therefore, the fact that higher maximum RS values for these metrics were recorded during the tournament, surpassing the maximum values previously achieved, appears to be associated with an increased risk of overuse or acute injury. In contrast, 75% of the athletes who did not sustain an injury (six out of eight) recorded higher maximum RS values prior to the tournament compared to during the tournament regarding ACC-DEC3. These results allow us to raise the possibility that it could be a good predictor of reduced injury risk.

Notably, this study presents several limitations that should be acknowledged. The retrospective design inherently carries a risk of bias, preventing the establishment of definitive causal relationships. While the RS approach enables a straightforward comparison of cumulative data across athletes, it does not provide the inferential strength of statistical significance testing. In terms of anthropometry and body composition, the reliance on self-reported parental height may have introduced bias, potentially compromising the accuracy of PAH estimations. The study’s single-center setting, combined with a relatively small sample size and the absence of a control group, limits its statistical power and external validity. Furthermore, the exclusion of goalkeepers and players not selected for the tournament may have introduced selection bias. The inclusion of athletes from only one elite U15 team may also restrict the generalizability of the findings to broader youth football populations. Additionally, important contextual variables such as preexisting conditions, playing positions, and tactical roles were not considered in the analysis, which could influence both performance and injury risk. Finally, the absence of internal load measurements restricts the study’s capacity to assess individual physiological responses to external workload.

## Conclusions

The findings of this study highlight three key factors that appear to be associated with an increased risk of injury in youth football players: maturation stage, external workload profiles, and congested competition schedules. All injuries recorded occurred in athletes who were in the circa- or post-PHV stages of maturation, reinforcing the vulnerability of players undergoing or having recently undergone accelerated growth. Furthermore, a substantial proportion of injured players presented higher RS values for TD, HSR, and ACC-DEC2 during the tournament when compared to their previously recorded maximums. This pattern suggests that sudden increases in accumulated external load may contribute significantly to injury risk. In contrast, most noninjured players exhibited higher RS values for ACC-DEC3 before the tournament than during it, indicating that progressive exposure to higher physical demands may serve as a protective factor. These results underline the importance of individualized load monitoring, especially in young athletes navigating critical phases of growth and development. Careful management of weekly microcycles and applied workloads emerges as a fundamental strategy to reduce injury risk and enhance player availability for training and match play.
